# The Knowledge and Value Basis of Private Forest Management in Sweden: Actual Knowledge, Confidence, and Value Priorities

**DOI:** 10.1007/s00267-020-01328-y

**Published:** 2020-07-21

**Authors:** Louise Eriksson, Clas Fries

**Affiliations:** 1 Department of Geography, Umeå University, SE-901 87 Umeå, Sweden; 2 Forest Unit, Swedish Forest Agency, Box 284, SE-901 06 Umeå, Sweden

**Keywords:** Forest management behavior, Production, Biodiversity, Recreation, Climate adaptation, Climate mitigation

## Abstract

With growing demands on forests, there is a need to understand the drivers of managing the forest for diverse objectives, such as production, recreation, and climate adaptation. The aim of this study was to examine the knowledge and value basis of forest management behaviors, including different management strategies and management inactivity, among private forest owners in Sweden. Different dimensions of knowledge (declarative and procedural knowledge, assessed in terms of objective and subjective knowledge measures) and value priorities (basic values and forest values), as well as the role of forest owner identity, were examined. The study was conducted by means of a postal questionnaire to a random sample of private forest owners in Sweden (*n* = 3000, response rate 43%). The distinctions between actual knowledge (objective knowledge), confidence (subjective knowledge), and value priorities, in addition to the hierarchical structure of how these factors are linked to management behaviors, proved to be valuable. Results revealed that different knowledge dimensions and value priorities were jointly important for forest management behaviors. In addition, the role of forest owner identity for management behaviors was confirmed. Insights from the study may be used to develop policy and outreach to private forest owners and thereby facilitate different forest functions in private forestry.

## Introduction

In 2015, the United Nations agreed on 17 Sustainable Development Goals to be achieved by 2030 and how forests are managed have implications for the attainment of several of these goals, for example clean water and sanitation (goal 6), affordable and clean energy (goal 7), climate action (goal 13), and life on land, including sustainable forest management (goal 15) (United Nations 2015). In this context, there are growing societal demands to use and manage the forest for production (e.g., timber), biodiversity conservation, carbon sequestration (through carbon storage or carbon substitution), and people’s health and wellbeing (Bellassen and Luyssaert 2014; Jactel et al. 2017; Lagergren and Jönsson 2017; Trivino et al. 2017). In addition, there is a need to reduce forests’ vulnerability to disturbances through, for example, climate change adaptation (Lindner et al. 2014). To facilitate diverse forest functions or multi-objective forestry, policy-makers, and practitioners need an understanding of the underlying basis for management decisions. In countries with a significant share of privately owned forests (e.g., the US, Germany, Sweden, and Finland), decisions concerning how to manage the forest are in the hands of family forest owners, also called individual private forest owners.

Previous research on forest owners has examined determinants of management activities, such as harvesting, the management of insects and invasive species, climate change adaptation, wildlife practices, and participation in different programs (e.g., concerning conservation). Results have revealed that structural characteristics relating to the owner and the forest (e.g., gender, age, forest type, size of forest, and distance from roads) are associated with management activities (e.g., Joshi and Arano 2009; Lidestav and Berg Lejon 2013; Silver et al. 2015; Coté et al. 2016; Aguilar et al. 2017; Thompson et al. 2017; Floress et al. 2019). In addition, social and psychological factors, such as social networks, personal experience, forest values and management objectives, subjective knowledge or awareness, and beliefs and attitudes have been found to be important for engagement in particular activities (Karppinen 2005; Joshi and Arano 2009; Blennow et al. 2012; Hendee and Flint 2013; Thompson and Hansen 2013; Põllumäe et al. 2014; Sagor and Becker 2014; Drescher et al. 2017; Kelly et al. 2017; Eriksson 2017, 2018b; Vulturius et al. 2018; Fischer 2019; Thorn et al. 2019). Even though knowledge has been found to play a role in forest management activities (e.g., Floress et al. 2019), and lack of knowledge is considered a significant barrier to achieving, in particular, new management aims such as climate change adaptation (Bissonnette et al. 2017; Sousa-Silva et al. 2018), the complexities associated with conceptualizing and measuring knowledge have largely been ignored. In addition, scarce attention has been given to the extent to which management decisions are formed based on knowledge as compared with other drivers. This study examined the knowledge and value basis of forest management in private forestry in Sweden. By using theoretically based concepts and carefully derived measures, and by comparing the drivers of different management strategies and management inactivity, the study contributes to an improved understanding of the underlying basis of forest management behaviors.

### Theoretical Background

The institutional and social context, with roots in history, has obvious implications for how private forest management is conducted (Andersson and Keskitalo 2018; Nichiforel et al. 2018). However, the heterogeneity among forest owners in the same setting suggests that the owners’ choice of management strategy cannot be sufficiently explained by contextual factors alone and a consideration of the psychological basis of management behaviors enables a more comprehensive understanding (Ingemarson et al. 2006; Vulturius et al. 2018).

#### Knowledge and forest management

There are diverse forms of knowledge, including science-based, but also systems of indigenous or local knowledge (The Intergovernmental Science-Policy Platform on Biodiversity and Ecosystem Services (IPBES) 2013; Hurlbert et al. 2019). In research on environmental behaviors, the individuals’ knowledge is considered important for the formation of perceptions and behaviors (Kaiser and Fuhrer 2003; Frick et al. 2004). However, there is a need to distinguish between knowledge types and different measures of knowledge (Vicente-Molina et al. 2013; Thorn and Bogner 2018). Declarative or system knowledge—e.g., how environmental systems or certain aspects of a system operate—can be differentiated from procedural or action-related knowledge, referring to knowledge of the specific actions that can be implemented to achieve a certain goal (Kaiser and Fuhrer 2003; Frick et al. 2004; Díaz-Siefer et al. 2015; Thorn and Bogner 2018). It is furthermore important to distinguish between objective (or actual) and subjective (or self-rated) assessments of knowledge (Shi et al. 2016). Whereas measures of objective knowledge employ knowledge questions (true/false or multiple choice), subjective knowledge represents a self-assessment of, for example, familiarity, awareness, or level of knowledge (Steele et al. 2006; Marzano et al. 2017), thus resembling the concept of self-efficacy, i.e., the belief in one’s own ability to act (Bandura 1977) (Geiger et al. 2019a).

In relation to forest owners, mainly measures of subjective knowledge tapping different knowledge dimensions have been employed, generally confirming an effect on management activities (Eggers et al. 2014; Fischer and Charnley 2012; Steele et al. 2006; Germain et al. 2014). For example, Eggers et al. (2014) showed that higher subjective knowledge about management was related to using a production-focused management approach. In addition, research on environmental behaviors shows that while subjective knowledge has been found to be more closely related to behavior, significant associations between objective knowledge and behavior have also been confirmed (Vicente-Molina et al. 2013; Díaz-Siefer et al. 2015; but see Ünal et al. 2017). Nevertheless, declarative objective knowledge have often been found to be indirectly related to behavior via other types of knowledge (e.g., procedural and effectiveness knowledge), attitudes, or intentions (Frick et al. 2004; Roczen et al. 2014; Kaiser and Fuhrer 2003; Nguyen et al. 2019).

#### Value priorities and forest management

Behaviors are also influenced by value priorities (Rohan 2000). The cognitive hierarchy model stipulates that cognitions can be arranged in a hierarchy from more general to specific cognitions (Fulton et al. 1996). On a general level, basic values transcend situations and act as general guiding principles for beliefs, attitudes, and behaviors. Schwartz’s (1992, 1994) value theory differentiates between two independent value dimensions; that is, values emphasizing self-interest (i.e., self-enhancement) versus others’ interests (i.e., self-transcendence, including altruistic and biospheric values) and values conveying an openness to new ideas (i.e., openness to change) versus maintaining the status quo (i.e., conservation). In addition, reasons why humans value forests have been labeled forest values, general forest beliefs or value orientations, highlighting for example production, recreation, ecological, aesthetic, and cultural forest values (e.g., McFarlane and Boxall 2003; Eriksson et al. 2013). In line with the cognitive hierarchy, associations between basic values and forest values have been confirmed, with self-transcendent values being positively correlated with ecological and recreation forest values, but negatively correlated with production values (Eriksson et al. 2013).

Evidence supports the importance of value priorities for management decisions. For example, forest owners emphasizing the interests of others and placing less emphasis on more traditional values tend to be more likely to participate in conservation programs (Drescher et al. 2017), and stronger production forest values, but also stronger ecological forest values, have been found to be associated with climate change adaptation (Eriksson 2018b). In addition, forest values have been incorporated into owner objectives, and the implementation of silvicultural measures, including thinning and harvesting, has been found to be higher among owners emphasizing timber and forest income than among other owners (Põllumäe et al. 2014; Joshi and Arano 2009). In contrast, an emphasis on amenity objectives has been found to be associated with lower levels of harvesting (Hendee and Flint 2013).

#### Forest owner identity and forest management

Self-identity refers to meanings attached to the self; and since people are motivated to act in accordance with how they view themselves, identity perceptions may influence behaviors (Burke and Stets 2009; Walton and Emmet Jones 2018). People generally have multiple identities that are more or less central to the overall self and vary in relevance across contexts. Self-identities may, for example, be based on group membership, such as forest ownership, with different meanings associated with the identity. In addition, identity perceptions may contain a social dimension reflecting the identification with a certain social group in conjunction with a differentiation from other groups. Since forest owners are heterogeneous, the meanings attached to a forest owner identity (FOI) may be diverse and cover sentiments such as being a multi-objective owner, a recreationist, economic centered, a farmer, an indifferent owner, a conservationist, multifunctional, or a self-employed owner, for example (Lawrence and Dandy 2014; Ní Dhubháin et al. 2007; Ficko et al. 2017; Feliciano et al. 2017). Studies suggest that perceptions of forest ownership may be incorporated as part of the owner’s identity (e.g., Bliss and Martin 1988; Lähdesmäki and Matilainen 2014; Kreye et al. 2018; Bergstèn et al. 2018), although scarce attention has been given to how different owner identities are associated with diverse management behaviors.

#### Conceptual framework

**Fig. 1:**
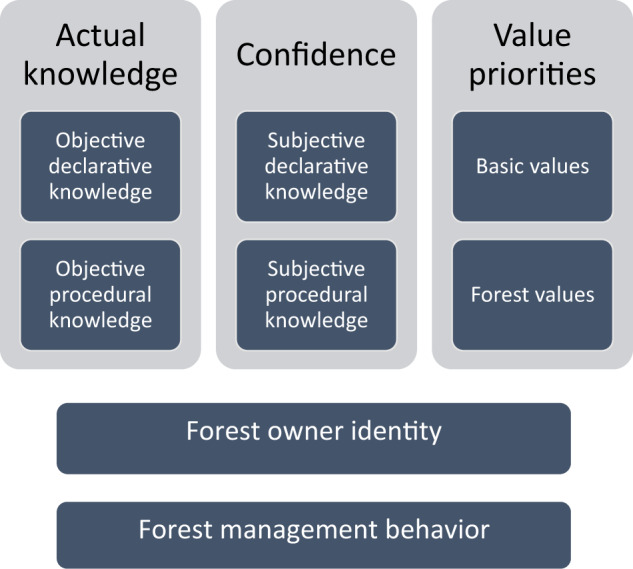
Conceptual framework of the hierarchically ordered knowledge and value basis of forest management behaviors

Based on the literature review, a conceptual framework depicting psychological drivers of forest management behaviors was developed, including knowledge and value priorities (see Fig. 1). Whereas these drivers have evolved concurrently over time and are thus interlinked, the conceptual distinctions will facilitate theoretical development and be useful for practice. A multidimensional concept of knowledge was employed (Shi et al. 2016), distinguishing between actual knowledge (objective knowledge) and confidence (subjective knowledge). The different concepts of knowledge and value priorities were considered to be hierarchically related to management behavior, with more general factors (i.e., declarative knowledge and basic values) being more distal predictors than behavioral specific factors (i.e., procedural knowledge and forest values) (Dietz et al. 1998; Gatersleben et al. 2017; Geiger et al. 2019b). Since forest owner identities reflect internalized perceptions (cf. Walton and Emmet Jones 2018), they should furthermore be more closely associated with management than knowledge and value priorities.

### The Present Study

Although knowledge and value priorities are both confirmed predictors of behaviors, their importance for forest management behaviors has not been compared and discussed. The aim of this study was to examine how actual knowledge, confidence and value priorities, as well as FOI, were associated with forest management behaviors among private forest owners in Sweden. Whereas previous studies of forest management activities have generally not compared the determinants of different management strategies (but see Joshi and Arano 2009), the present study explored predictors of different types of management behaviors, including management for production, biodiversity, recreation, climate adaptation, and climate mitigation. With thought to the changes in forest ownership in many Western countries, e.g., more absentee owners and fewer owners relying on their forest for income (Hogl et al. 2005; Ficko et al. 2017; Weiss et al. 2019), the determinants of management inactivity were also explored. Overall, the study examined: (1) forest management behaviors (i.e., frequency of engaging in different management strategies, including management inactivity, and relations between strategies); (2) structural correlates of forest management behaviors, including gender, age, education, size of forest holding, residency, place, and region; and (3) the importance of actual knowledge, confidence, value priorities, and FOI for forest management behaviors. Based on the conceptual framework, actual knowledge, confidence, and value priorities should all be associated with management behaviors. In addition, the psychological drivers were expected to be hierarchically ordered in relation to management behaviors, with more general concepts being more distant predictors than specific concepts. Whereas confidence is a key determinant of a broad range of behaviors (cf. Ajzen 2002), and both confidence and value priorities have been found to be relevant for forest management (Eggers et al. 2014; Eriksson 2018b), actual knowledge has been given less attention. Thus, no hypotheses regarding the relative importance of the different knowledge dimensions and value priorities for management behaviors were generated.

## Materials and Methods

### Study Area

Close to 70% of the land area in Sweden is covered by forests, and coniferous trees, primarily Norway Spruce and Scots Pine, are the main tree species (Swedish University of Agricultural Science [SLU] 2018). The majority of the forest in Sweden is privately owned, with ~50% owned by around 330,000 individual private forest owners (Swedish Forest Agency [SFA] 2014). The environmental and production objectives in the Swedish forest policy are considered equally important, and forest is to be used for a variety of different purposes, including adapting it to climate change and using it for climate mitigation (Swedish Gov. Bill 2007/08:108). Nevertheless, the forest is a significant economic asset, with its large production of roundwood and sawnwood (Eurostat 2017), and studies have shown that the forestry culture in Sweden is dominated by production objectives (e.g., Andersson and Keskitalo 2018). Whereas management was regulated in detail before 1993, with several mandatory silvicultural measures preventing management inactivity, only a few obligatory measures remain (e.g., regeneration after clear felling) and the forest owners enjoy a great degree of freedom (Bush 2010). In this context, information and advice are considered important tools to achieve the goals of the forest policy (Johansson and Keskitalo 2014).

### Respondents

A postal questionnaire to a randomly selected sample of individual private forest owners in Sweden, aged 20–80 years and owning more than 5 ha of forest land, was conducted by a survey company (Attityd i Karlstad AB) in the autumn of 2018. After two reminders, the response rate was 43% (*n* = 1251). The sample contained 19% women and the mean age was 62 years (SD = 11). Almost a third of the respondents had a university degree (31%) and about half, 52%, were resident owners. The mean area of productive forest was 92 ha (SD = 260). Whereas differences between the population and sample were minor, the sample did contain fewer women, young owners, and owners with small forest properties. Hence, calibrated weights were used in the analyses to control for these deviations.

### Questionnaire

Information on gender, age, size of forest holding, and region where the forest property was located were taken from the owner register. Background questions included, for example, education, whether the owner was resident or nonresident, and whether the owner lived in an urban or a rural area. In addition, actual knowledge, confidence, value priorities, FOI, and forest management behaviors were assessed in the questionnaire. Means, standard deviations, and internal reliability (alpha) for the psychological predictors are displayed in the Appendix (Table A1).

*Actual knowledge* was examined using objective knowledge scales reflecting declarative knowledge about the forest in Sweden more generally and procedural knowledge in relation to each of the five management types. The construction of the knowledge scales was guided by research on how to measure knowledge (considering, e.g., difficulty levels) (Frick et al. 2004; Díaz-Siefer et al. 2015) and forest facts, also involving a forest management expert at the SFA. The battery of questions at Skogskunskap.se (a web portal with facts about forests) was used as inspiration for some of the questions. The initial set of questions was pretested by a group of forest owners, answering the questions and evaluating and commenting on their clarity, etc., as well as rating them on a three-point scale: easy, medium, or difficult. After revisions, six questions reflecting general knowledge (two for each of the three difficulty levels) and 20 questions reflecting the five types of procedural knowledge (including one easy, two medium, and one difficult question on each of the scales) were included in the questionnaire. A multi-response format was used, with three to six correct response options for each question. The questions are available from the authors upon request. Answers were coded in three categories—0 = wrong, 0.5 = partly correct, and 1 = correct—resulting in a scale from 0 to 6 on general knowledge and a scale from 0 to 4 on procedural knowledge.

*Confidence* was examined using subjective knowledge measures about the forest in general and about each of the five management types. Based on previous research on subjective knowledge (McFarlane and Watson 2008), the owners were asked about how much knowledge they considered themselves to have about the following: general knowledge about forests in Sweden (e.g., tree species, damage, and ownership conditions); forest management aiming for good forest growth; forest management used to preserve biodiversity; forest management contributing to an attractive recreation forest; forest management aiming to use the forest for climate mitigation; and forest management adjusted to a warmer climate. Answers were provided on a four-point scale (1 = no knowledge at all, 2 = a little knowledge, 3 = certain knowledge, 4 = extensive knowledge).

*Value priorities* in terms of basic values and three types of forest values were assessed. Based on Schwartz’s (1992, 1994) value theory and the distinction between altruistic, biospheric, and egoistic value orientations (de Groot and Steg 2008), the following basic values were assessed: openness (five items), conservation (five items), self-enhancement (SE) (five items), and self-transcendence (ST) (including altruism (Alt) (four items) and biospheric (Bio) (four items)). The respondents were asked to indicate how important each value was as a guiding principle in their life, with responses provided on a nine-point scale (−1 = opposed to my values, 0 = not important, 3 = important, 6 = very important, and 7 = extremely important). Before combining the values into higher-order value types, scale use differences were controlled for by mean centering the higher-order value score as suggested by Schwartz. A confirmatory factor analyses with varimax rotation of the higher-order value indexes (62% explained variance) confirmed a two-factor model. Because of the relevance of the SE–ST scale for environmental behaviors (Stern 2000), only the factor scores based on this dimension were included in the final analyses. To assess forest values, the owners were asked how important they believed production (e.g., timber or biofuel), the possibilities for recreation for humans, and biodiversity (diversity in plant and animal life) were in their own forest and in the Swedish forest in general, respectively (cf. Eriksson 2018a). Answers were provided on a seven-point scale (1 = not at all important, 7 = very important) and index variables were created by calculating the means of the two items for each forest value scale.

*Forest owner identity*, in terms of the meanings attached to the owners’ self- and social forest owner identities, as well as centrality, was assessed. The owners were asked about the extent to which they agreed with statements reflecting how they use, manage, and perceive their forest (self-identity), and the extent to which they identified with different types of other forest owners (social identity). Four owner identities were measured, reflecting primary ways in which the forest may be perceived and used, relationships with other owners, and the forest itself; i.e., production and private asset (production/private), consumption of noneconomic values and public resource (consumption/public), connections with other owners (social), and detachment from forest (distant). Answers were provided on a five-point scale (1 = totally disagree, 5 = totally agree). Since the identity scales had not been previously tested, alpha values guided their revisions. Removing any item from the Social FOI or the Distant FOI did not increase the internal reliability. However, when one item was excluded from the two remaining scales, the alpha values increased slightly. A measure of centrality of the forest to the owners was developed measuring positive emotions, the possession-self link, and importance (six items) (cf. Ferraro et al. 2011). Answers were provided on a five-point response scale (1 = totally disagree, 5 = totally agree) and the mean of the items was used to create a centrality index. The distant and the consumption/public FOI scales displayed a somewhat low reliability (*α* = 0.65 and *α* = 0.62, respectively) and this should be considered when interpreting results. The FOI items are provided in the Appendix (see Table A2).

*Forest management behavior* included the frequency of implementing different management strategies and management inactivity. Production, biodiversity, recreation, and climate adaptation management behaviors were examined by means of four items each, and climate mitigation (in terms of substitution) was assessed using three items. The owners were asked about how often they had used different strategies in their forest and the answers were provided on a five-point scale (1 = never, 2 = seldom, 3 = sometimes, 4 = often, 5 = always) (see the Appendix (Table A3) for the list of included management strategies). Subsequently, the sum of the included strategies was calculated, resulting in a scale from 1 to 20 for all management strategies except climate mitigation (substitution), which had a scale from 1 to 15. To assess management inactivity, the owners were asked to indicate whether they had refrained from implementing any forest management measure during the last 10 years.

### Analyses

For data analyses, SPSS Statistics 24 was utilized (IBM corp. 2016). First, forest management behaviors were described via means and standard deviations for the five management strategies and the percentage of owners displaying management inactivity. In addition, correlations between management behaviors were analyzed using Pearson’s *r* for the management strategies and point-biserial correlation for management inactivity.

Second, linear regression analyses were used to examine relations between structural characteristics and the different management strategies, and a binary logistic regression analysis was employed to analyze relations between structural characteristics and management inactivity. Gender (dummy: 1 = female), age, education (dummy: 1 = University degree), size of forest holding, residency (dummy: 1 = resident owner), place (dummy: 1 = urban), and region (dummy: 1 = South region corresponding to the organizational setup of the SFA) were included as independent variables. Dependent variables were management strategies (i.e., frequency of engaging in production, biodiversity, recreation, adaptation, and mitigation (substitution) management) and management inactivity, respectively.

Third, the importance of knowledge, value priorities, and FOI for forest management behaviors was examined by means of hierarchical regression analyses in three steps. Linear regression analyses were used to examine predictors of the different forest management strategies, and a binary logistic regression analysis was employed to analyze predictors of management inactivity. In the first step, general variables (i.e., declarative objective knowledge, declarative subjective knowledge, and basic values) were included in the analyses of both forest management strategies and management inactivity. In the second step, the more specific knowledge and value variables (i.e., procedural objective knowledge, procedural subjective knowledge, and forest values) were added. In the analyses of management strategies, one procedural knowledge measure and one forest value scale were examined in relation to each strategy (e.g., procedural objective knowledge of production in relation to production management), except in relation to adaptation and mitigation (substitution) management. Since these strategies may be motivated by diverse forest values (Eriksson 2018a), both production and biodiversity forest values were included. In the analysis of management inactivity, all measures of actual knowledge, confidence, and forest values were used as predictors. Finally, in an explorative manner, the Social FOI, the Distant FOI, and centrality were included in relation to all management strategies. In addition, Production/private FOI was included in relation to production management, consumption/public FOI in relation to biodiversity and recreation management, and both these FOIs were examined in relation to adaptation and mitigation (substitution) management. The full set of FOIs was included as predictors in the third step of the analysis of management inactivity.

## Results

### Forest Management Behaviors

Descriptives and the associations between different measures of forest management behaviors are displayed in Table 1. Whereas the owners did not frequently engage in mitigation (substitution) management, the means for the remaining strategies were close to the midpoint of the scale. About one fourth of the respondents had not engaged in any forest management activities the last 10 years. The positive correlations between the strategies suggest that owners implementing one type of strategy were more likely to implement the other strategies. A strong positive correlation was found between climate adaptation management and both biodiversity and recreation management. In contrast, biodiversity management and climate mitigation (substitution) management displayed the weakest correlation. Management inactivity displayed the strongest negative correlation with production management, indicating that inactive owners were the least likely to implement production-oriented activities.

**Table 1:** Descriptives and bivariate correlations for forest management behaviors

	Production^a^	Biodiversity^a^	Recreation^a^	Adaptation^a^	Mitigation (substitution)^b^	Management inactivity
Production	*M* = 10.27, SD = 3.44					
Biodiversity	0.27***	*M* = 11.36, SD = 2.79				
Recreation	0.26***	0.48***	*M* = 10.74, SD = 3.65			
Adaptation	0.43***	0.53***	0.54***	*M* = 10.98, SD = 3.74		
Mitigation	0.41***	0.21***	0.31***	0.39***	*M* = 4.79, SD = 1.85	
Management inactivity	−0.44***	−0.24***	−0.17***	−0.33***	−0.21***	25.1%

***p < 0.001

^a^Scale 1–20, ranging from never to always

^b^Scale 1–15, ranging from never to always

### Structural Characteristics and Forest Management Behaviors

Results from the regression analyses of how structural characteristics are related to forest management behaviors are displayed in Table 2. There was no evidence of collinearity, since no VIF value exceeded 1.303 in any of the models. Results revealed that male owners, owners with larger forest holdings, and owners in the south region had implemented all the management strategies to a greater extent and were less likely to be inactive compared to their counterparts. Older owners, compared with younger owners, had more frequently engaged in production, biodiversity, and mitigation (substitution) management. In addition, owners with a university degree had more often implemented biodiversity management, resident owners had more often implemented mitigation (substitution) management, and rural owners had more often employed recreation and adaptation management, compared to their counterparts. The structural factors and forest characteristics explained between 5 and 8% of the variance in the different management strategies.

**Table 2:** Structural characteristics as predictors of forest management strategies and management inactivity

	Production		Biodiversity		Recreation		Adaptation		Mitigation (substitution)		Management inactivity		
	B (SE)	*β*	B (SE)	*β*	B (SE)	*β*	B (SE)	*β*	B (SE)	*β*	B (SE)	Wald	Exp (B)
Gender (1 = female)	−0.0814 (0.240)	−0.10***	−0.487 (0.197)	−0.08*	−1.356 (0.256)	−0.16***	−1.134 (0.264)	−0.13***	−0.537 (0.129)	−0.13***	0.410 (0.171)	5.785*	1.507
Age	0.037 (0.009)	0.12***	0.026 (0.007)	0.11***	0.009 (0.010)	0.03	0.018 (0.010)	0.06	0.010 (0.005)	0.06*	0.010 (0.007)	2.293	1.010
Education (1 = University)	0.262 (0.231)	0.04	0.925 (0.190)	0.16***	0.456 (0.246)	0.06	0.435 (0.253)	0.06	0.144 (0.124)	0.04	−0.147 (0.173)	0.724	0.863
Size of forest holding	0.004 (0.001)	0.22***	0.001 (0.000)	0.07*	0.001 (0.001)	0.06*	0.002 (0.001)	0.10***	0.001 (0.000)	0.14***	−0.026 (0.003)	64.930***	0.974
Residency (1 = resident)	0.008 (0.221)	0.00	0.175 (0.182)	0.03	0.278 (0.236)	0.04	0.377 (0.242)	0.05	0.251 (0.118)	0.07*	0.048 (0.161)	0.089	1.049
Place (1 = urban)	−0.343 (0.299)	−0.04	−0.423 (0.246)	−0.06	−1.499 (0.321)	−0.16***	−0.886 (0.328)	−0.09**	−0.262 (0.161)	−0.05	−0.384 (0.225)	2.921	0.681
Region (1 = South)	0.558 (0.208)	0.08**	0.818 (0.171)	0.15***	0.822 (0.222)	0.11***	1.141 (0.228)	0.15***	0.686 (0.112)	0.18***	−0.437 (0.157)	7.709**	0.646
Constant	7.744 (0.595)		9.243 (0.489)		10.082 (0.635)		9.459 (0.652)		3.843 (0.319)		−0.731 (0.451)	2.618	0.482
* R*^2^	0.08***		0.06***		0.08***		0.07***		0.09***		na		
Adj *R*^2^	0.07***		0.05***		0.07***		0.06***		0.08***		na		
Nagelkerke	na		na		na		na		na		0.201		
−2 Log likelihood	na		na		na		na		na		1100.71		

*na* not applicable

*p < 0.05; **p < 0.01; ***p < 0.001

### Psychological Drivers of Forest Management Behaviors

The hierarchical analyses of psychological drivers of management behaviors are displayed in Table 3 for the forest management strategies, and in Table 4 for management inactivity. The models of forest management strategies displayed no evidence of collinearity (no VIF value exceeded 2.342). Among the general variables in the first step, declarative subjective knowledge and basic values were significant predictors of all management strategies except recreation management, where only declarative subjective knowledge was significant. In the second step the beta weights for the general variables decreased, although declarative subjective knowledge was still significant in relation to all management strategies except production management, and basic values were significant in relation to production and mitigation (substitution) management. Whereas procedural objective knowledge was a significant predictor in relation to production, biodiversity, and adaptation management, procedural subjective knowledge, and forest values were significant in relation to all management strategies. In the third step, the beta weights of the more general variables decreased even further; however, procedural subjective knowledge was still significant in relation to all management strategies. In addition, basic values and production values significantly determined production management, and production values were a significant predictor of mitigation (substitution) management. Whereas the Production/private FOI predicted production and mitigation (substitution) management, the consumption/public FOI predicted biodiversity, recreation, and adaptation management. In addition, the social FOI was positively linked to all management strategies and the Distant FOI was negatively associated with production, recreation, and adaptation management. Centrality of the identity was positively correlated with recreation and adaptation management. Two variables displayed reversed signs in the final step of the analyses (declarative subjective knowledge in relation to production management and declarative objective knowledge in relation to adaptation management), indicating that they act as suppressor variables in these models. In each step of the analyses, the explained variance increased significantly. Whereas the predictors explained a relatively low level of variance in mitigation (substitution) management, they were more important for production and adaptation management.

**Table 3:** Hierarchical linear regression analyses of forest management strategies in three steps: (1) general variables (declarative knowledge and basic values), (2) specific variables (procedural knowledge and forest values), (3) forest owner identities

	Production		Biodiversity		Recreation		Adaptation		Mitigation (substitution)	
	B (SE)	*β*	B (SE)	*β*	B (SE)	*β*	B (SE)	*β*	B (SE)	*β*
Step 1										
DEC-OBJ	0.066 (0.079)	0.02	0.123 (0.065)	0.06	0.014 (0.084)	0.01	0.096 (0.082)	0.03	−0.020 (0.044)	−0.01
DEC-SUBJ	1.445 (0.141)	0.30***	1.088 (0.116)	0.28***	1.444 (0.151)	0.28***	2.168 (0.148)	0.41***	0.535 (0.079)	0.20***
SE–ST	−0.460 (0.096)	−0.14***	0.302 (0.079)	0.11***	0.111 (0.104)	0.03	0.212 (0.102)	0.06*	−0.254 (0.054)	−0.14***
Constant	6.131 (0.448)		7.935 (0.372)		6.774 (0.479)		4.724 (0.472)		3.454 (0.252)	
*R*^2^	0.11***		0.10***		0.08***		0.18***		0.06***	
Adj *R*^2^	0.11***		0.09***		0.08***		0.18***		0.06***	
Step 2										
DEC-OBJ	−0.073 (0.075)	−0.03	0.061 (0.064)	0.03	−0.002 (0.081)	0.00	−0.101 (0.081)	−0.03	−0.019 (0.045)	−0.01
DEC-SUBJ	−0.247 (0.179)	−0.05	0.301 (0.140)	0.08*	0.611 (0.165)	0.12***	1.157 (0.168)	0.22***	0.258 (0.094)	0.10**
SE–ST	−0.305 (0.088)	−0.09***	0.105 (0.083)	0.04	−0.034 (0.103)	−0.01	0.143 (0.108)	0.04	−0.216 (0.060)	−0.12***
PROC-OBJ	0.374 (0.132)	0.09**	0.279 (0.093)	0.09**	−0.231 (0.133)	−0.05	0.590 (0.126)	0.14***	−0.143 (0.077)	−0.06
PROC-SUBJ	1.630 (0.172)	0.36***	1.129 (0.132)	0.30***	1.692 (0.152)	0.36***	1.222 (0.154)	0.24***	0.431 (0.086)	0.17***
PROD VALUES	0.695 (0.072)	0.27***	na	na	na	na	0.373 (0.080)	0.13***	0.145 (0.044)	0.10***
BIO VALUES	na	na	0.162 (0.064)	0.08*	na	na	0.244 (0.082)	0.09**	−0.028 (0.046)	−0.02
REC VALUES	na	na	na	na	0.145 (0.072)	0.06*	na	na	na	na
Constant	2.000 (0.497)		5.953 (0.495)		4.744 (0.606)		0.731 (0.659)		2.897 (0.378)	
R^2^	0.28***		0.17***		0.19***		0.27***		0.10***	
Adj R^2^	0.27***		0.17***		0.19***		0.27***		0.09***	
∆ *R*²	0.16***		0.08***		0.11***		0.09***		0.03***	
Step 3										
DEC-OBJ	−0.125 (0.071)	−0.05	0.019 (0.063)	0.01	−0.135 (0.078)	−0.05	−0.194 (0.076)	−0.07*	−0.046 (0.044)	−0.03
DEC-SUBJ	−0.472 (0.170)	−0.10**	0.134 (0.142)	0.03	0.060 (0.166)	0.01	0.630 (0.164)	0.12***	0.107 (0.096)	0.04
SE–ST	−0.203 (0.084)	−0.06*	0.140 (0.082)	0.05	−0.029 (0.097)	−0.01	0.167 (0.101)	0.05	−0.192 (0.059)	−0.10***
PROC-OBJ	0.194 (0.126)	0.04	0.248 (0.093)	0.08**	−0.186 (0.126)	−0.04	0.470 (0.119)	0.11***	−0.100 (0.076)	−0.04
PROC-SUBJ	1.154 (0.169)	0.25***	0.965 (0.134)	0.26***	1.349 (0.147)	0.29***	0.907 (0.148)	0.18***	0.330 (0.085)	0.13***
PROD VALUES	0.371 (0.074)	0.14***	na	na	na	na	0.105 (0.082)	0.04	0.014 (0.048)	0.01
BIO VALUES	na	na	0.035 (0.067)	0.02	na	na	0.066 (0.083)	0.02	−0.019 (0.048)	−0.01
REC VALUES	na	na	na	na	0.016 (0.071)	0.01	na	na	na	na
PROD_PRIVATE FOI	1.108 (0.137)	0.27***	na	na	na	na	0.293 (0.151)	0.07	0.408 (0.088)	0.18***
CON_PUBLIC FOI	na	na	0.575 (0.131)	0.15***	1.028 (0.160)	0.21***	0.970 (0.159)	0.19***	0.024 (0.093)	0.01
SOCIAL FOI	0.384 (0.098)	0.12***	0.229 (0.082)	0.09**	0.289 (0.100)	0.09**	0.238 (0.106)	0.07*	0.184 (0.061)	0.11**
DISTANT FOI	−0.554 (0.142)	−0.11***	0.006 (0.127)	0.00	−0.548 (0.156)	−0.10***	−0.728 (0.153)	−0.14***	−0.018 (0.089)	−0.01
CENTRAL FOI	−0.141 (0.125)	−0.03	0.044 (0.121)	0.01	0.352 (0.147)	0.08*	0.305 (0.145)	0.07*	−0.044 (0.084)	−0.02
Constant	3.839 (0.764)		4.903 (0.702)		3.318 (0.865)		1.133 (0.874)		2.727 (0.527)	
*R*^2^	0.36***		0.20***		0.28***		0.36***		0.14***	
Adj *R*^2^	0.36***		0.20***		0.28***		0.36***		0.13***	

*DEC-OBJ* declarative objective knowledge, *PROC-OBJ* procedural objective knowledge (*PROD* production, *BIO* biodiversity, *REC* recreation, *MIT* mitigation, and *ADAPT* adaptation). *DEC-SUBJ* declarative subjective knowledge, *PROC-SUBJ* procedural subjective knowledge (*PROD* production, *BIO* biodiversity, *REC* recreation, *ADAPT* adaptation, and *MIT* mitigation). *SE–ST* self-enhancement versus self-transcendence, *PROD VALUE* production forest values, *BIO VALUE* biodiversity forest values, *REC VALUE* recreation forest values. *PROD PRIVATE FOI* production/private forest owner identity, *CON PUBLIC FOI* consumption/public forest owner identity, *SOCIAL FOI* social forest owner identity, *DISTANT FOI* distant forest owner identity, *CENTRAL FOI* central forest owner identity. *na* not applicable

*p < 0.05; **p  < 0.01; ***p < 0.001

**Table 4:** Hierarchical binary logistic regression analysis of management inactivity in three steps: (1) general variables (declarative knowledge and basic values), (2) specific variables (procedural knowledge and forest values), (3) forest owner identities

	Step 1			Step 2			Step 3		
	B (SE)	Wald	Exp (B)	B (SE)	Wald	Exp (B)	B (SE)	Wald	Exp (B)
DEC-OBJ	−0.200 (0.059)	11.302***	0.819	−0.111 (0.067)	2.702	0.895	−0.073 (0.073)	0.995	0.930
DEC-SUBJ	−0.861 (0.109)	62.195***	0.423	−0.195 (0.168)	1.356	0.822	−0.076 (0.179)	0.179	0.927
SE–ST	0.077 (0.072)	1.156	1.080	0.011 (0.086)	0.016	1.011	−0.043 (0.090)	0.232	0.958
PROC-OBJ PROD				−0.433 (0.125)	11.898***	0.649	−0.332 (0.134)	6.088*	0.718
PROC-OBJ BIO				−0.371 (0.104)	12.652***	0.690	−0.353 (0.109)	10.447***	0.703
PROC-OBJ REC				−0.320 (0.111)	8.268**	0.726	−0.344 (0.117)	8.678**	0.709
PROC-OBJ ADAPT				0.440 (0.116)	14.350***	1.552	0.531 (0.124)	18.271***	1.701
PROC-OBJ MIT				0.051 (0.121)	0.176	1.052	−0.022 (0.129)	0.030	0.978
PROC-SUBJ PROD				−0.649 (0.175)	13.768***	0.523	−0.426 (0.187)	5.207*	0.653
PROC-SUBJ BIO				0.197 (0.183)	1.158	1.218	0.277 (0.197)	1.987	1.320
PROC-SUBJ REC				0.077 (0.155)	0.247	1.080	0.089 (0.165)	0.294	1.094
PROC-SUBJ ADAPT				−0.336 (0.164)	4.199*	0.715	−0.218 (0.173)	1.584	0.804
PROC-SUBJ MIT				0.082 (0.176)	0.216	1.085	0.075 (0.188)	0.160	1.078
PROD VALUES				−0.415 (0.065)	40.482***	0.660	−0.225 (0.072)	9.728**	0.798
BIO VALUES				0.009 (0.082)	0.011	1.009	0.029 (0.090)	0.103	1.029
REC VALUES				0.004 (0.075)	0.003	1.004	−0.031 (0.080)	0.149	0.970
PROD_PRIVATE FOI							−0.492 (0.150)	10.751***	0.612
CON_PUBLIC FOI							0.057 (0.146)	0.154	1.059
SOCIAL FOI							−0.613 (0.113)	29.252***	0.542
DISTANT FOI							0.352 (0.137)	6.609**	1.422
CENTRAL FOI							−0.150 (0.129)	1.346	0.861
Constant	1.908 (0.339)	31.635***	6.739	4.683 (0.619)	57.177***	108.04	4.289 (0.852)	25.331***	72.905
Nagelkerke *R* square	0.12			0.27			0.36		
−2 Log likelihood	1134.25			1010.69			921.11		

*DEC-OBJ* declarative objective knowledge, *PROC-OBJ* procedural objective knowledge (*PROD* production, *BIO* biodiversity, *REC* recreation, *MIT* mitigation, and *ADAPT* adaptation). *DEC-SUBJ* declarative subjective knowledge, *PROC-SUBJ* procedural subjective knowledge (*PROD* production, *BIO* biodiversity, *REC* recreation, *ADAPT* adaptation, and *MIT* mitigation). *SE–ST* self-enhancement versus self-transcendence, *PROD VALUE* production forest values, *BIO VALUE* biodiversity forest values, *REC VALUE* recreation forest values. *PROD PRIVATE FOI* production/private forest owner identity, *CON PUBLIC FOI* consumption/public forest owner identity, *SOCIAL FOI* social forest owner identity, *DISTANT FOI* distant forest owner identity, *CENTRAL FOI* central forest owner identity

*p < 0.05, **p < 0.01, ***p < 0.001

The analyses of forest management inactivity showed that, in the first step, declarative objective and subjective knowledge were significant predictors but basic values were not. In subsequent steps, however, none of these variables remained significant. In the second step, procedural objective knowledge of all forest management strategies except mitigation was significant, as was procedural subjective knowledge of production and adaptation. In addition, production forest values were a significant predictor of management inactivity. Hence, whereas several different types of objective knowledge were significant predictors of management inactivity, fewer measures of subjective knowledge, and forest values were. The same measures of procedural objective and subjective knowledge, in addition to production forest values, remained significant in the third step. In addition, a weaker Production/private FOI and a weaker Social FOI, but a stronger Distant FOI, were associated with management inactivity. Whereas less knowledge and weaker production forest values were generally associated with a higher probability of not managing the forest, a higher level of procedural objective knowledge of adaptation management was associated with management inactivity. The full model was significantly better in explaining management inactivity compared to the models with variables reflecting knowledge and value priorities.

## Discussion

This study confirms that different knowledge dimensions and value priorities are jointly important for forest management behaviors, adding to the ongoing discussion of how knowledge versus values influence behaviors (cf. Shi et al. 2016; Ünal et al. 2017; Bamberg and Möser 2007). These results are timely, and have implications for the future governance of private forestry, given the diverse demands on forest use and management (Lagergren and Jönsson 2017). The study may further spur an interest for a novel knowledge approach in the context of adaptive management where knowledge is considered a key asset (Fabricius and Cundill 2014).

The differential effects of knowledge on management behaviors shown in this study indicate that it is problematic to simply refer to knowledge as an important determinant (cf. Floress et al. 2019). Comparable to previous research in the environmental domain (e.g., Frick et al. 2004), the results generally supported the more remote role of declarative compared to procedural knowledge in relation to management behaviors, including management inactivity. Furthermore, procedural subjective knowledge was a significant predictor even in the final step of the analyses in all the models, whereas results for procedural objective knowledge were less consistent. Hence, in line with previous studies (Vicente-Molina et al. 2013; Eggers et al. 2014), the importance of subjective knowledge in relation to diverse management strategies was supported. However, this study could not confirm that subjective knowledge was more important for management inactivity. Overall, the results verified a positive association between knowledge and forest management behaviors, irrespective of type of knowledge, with one exception: whereas being more knowledgeable about climate adaptation was associated with more frequently implementing adaptation measures (e.g., increasing the share of mixed and broadleaved forest), it was also associated with management inactivity. A less proactive approach to the risk of future damages in forests has been found among less engaged owners (Gan et al. 2015). However, not implementing certain management measures may also reflect a willingness to rely on the forest’s own ability to adapt through evolutionary processes (i.e., passive adaptation) (Keskitalo et al. 2016; Hagerman and Pilai 2018).

By confirming the different value basis of production and mitigation (substitution) management versus biodiversity management, and the dual value basis of climate adaptation, this study further expands on how the owners’ emphasis on SE versus ST values are relevant for forest management behaviors (e.g., Dreschel et al. 2017; Eriksson 2018a, b). As expected, the importance of basic values generally decreased after the inclusion of more specific variables, although not in relation to production and mitigation (substitution) management. Results suggest that value priorities (i.e., production values) are particularly important for production management. Since situational constraints may prevent values from having an impact on actual behavior (Steg et al. 2014; see also Põllumäe et al. 2014), the weaker effect of non-production values on management behaviors in this sample may stem from the production-oriented focus of forestry in Sweden (Keskitalo et al. 2016; Andersson and Keskitalo 2018). Even though there are no regulatory barriers to alternative management, the production forestry culture may, through normative processes, facilitate production and discourage alternatives. The study further revealed that the internalization of core interests (i.e., production versus consumption) in terms of forest owner identities was relevant for management behaviors. The results are generally in line with depictions of identity as a mediator between value priorities and behaviors (cf. Gatersleben et al. 2017). Previous studies have confirmed that social factors play an important role for forest owners’ behaviors (Ruseva et al. 2014; Eriksson 2018a). This study advances this line of research by outlining a potential psychological mechanism for how the social context may influence behaviors. Owners interacting with other owners in various ways are likely to internalize perceptions of being a social owner, and this connectedness to others may in turn facilitate an active management approach. Since the Social FOI was positively associated with diverse management strategies, it is worth pointing out that the owners’ networks seem to facilitate different management objectives, despite the emphasis on production in the Swedish forestry context.

Active or passive forest management approaches may be advocated depending on, for example, the purpose of the management, such as maximizing certain forest functions or multifunctionality (e.g., Hagerman and Pilai 2018; Cruz-Alonso et al. 2019; White and Long 2019; Williams and Powers 2019). Whereas this study showed that certain structural characteristics were associated with management inactivity (owning a smaller forest holding, being female, and owning forest in the north and middle regions in Sweden) (cf. Eriksson 2018b), results further revealed that an overall lesser focus on production (knowledge and value priorities) and a lower identification with other owners also characterized management inactivity. Potentially reflecting the production focus in this context but also that a norm of active management is remaining in Sweden despite the lower regulatory demands on management (Bush 2010). Nonresident owners, and owners living in an urban context, were not more likely to display inactivity, thus indicating that spatial distance to the forest do not necessarily mean a lower involvement in management (cf. Huff et al. 2017). However, worth noticing is the importance of different competences for an active forest management approach (i.e., more actual knowledge of biodiversity, recreation, and production management), showing that a broad range of skills is needed for owners to actively manage their forest.

When interpreting the results of this study, some limitations should be considered. Although a representative sample of forest owners in Sweden was surveyed and calibrated weights were used to avoid biases, active forest owners are likely overrepresented in the sample since they are likely more interested in the topic of the study. The measures were carefully developed and the internal reliability was generally good. However, despite being significant predictors, some of the scales measuring FOI displayed low reliability, indicating a need to develop these measures, and validate them in future research. The study was theoretically based, but since cross-sectional data cannot support causality, experimental evidence is needed to confirm the effect of independent variables on management behaviors (e.g., by exploring how different knowledge interventions influence management behaviors). While an overall assessment of the importance of psychological predictors for management behaviors is frequently missing in previous studies, the level of explained variance of production and climate adaptation management behaviors was comparable to results by Karppinen (2005) and Dreschel et al. (2017), and the level of explained variance in biodiversity and recreation management was equivalent to that in the study by Eriksson (2017). To develop the understanding of the individual drivers of forest management behaviors, it may be valuable to consider interactions between knowledge and value priorities in future research (e.g., values may be important for the acquisition of knowledge) (Thorn and Bogner 2018). In addition, to determine the generality of the results, there is a need to explore how different types of knowledge (including local and traditional knowledge) and value priorities, as well as different indicators of management behaviors, are related in diverse samples and contexts.

## Conclusion

This study distinguished between actual knowledge, confidence, and value priorities, and confirms the independent effects of these factors along with forest owner identities on management behaviors among forest owners. Its results contribute novel insights for the understanding of the individual drivers of forest management behaviors, and the approach may be drawn upon to advance the understanding of the psychological basis of natural resource management more generally. In addition, the study has implications for governance. For example, more actual forest knowledge may not only lead to more informed management decisions; this study suggests that increasing particularly procedural knowledge of different management strategies may facilitate management. Although supporting social networks and increasing actual knowledge of different management strategies are likely to encourage a more active management approach, boosting the owner’s confidence to implement specific management strategies (e.g., production or recreation) is important in order to facilitate particular management aims. Since a more varied forest may be more resistant to damage (e.g., Jactel et al. 2017), there may be a need to ensure that cultural and social factors do not prevent the diversity in owners’ value profiles from being realized in their management practices. Moreover, by increasing the salience of specific owner identities in outreach to owners, specific forest functions may be encouraged.

## Supplementary information

Appendix

## Data Availability

The dataset analysed during the current study is available from the corresponding author upon reasonable request after the completion of the project.
