# Supplementary material for: The Knowledge and Value Basis of Private Forest Management in Sweden: Actual Knowledge, Confidence, and Value Priorities
Source: Environ Manage. 2020 Jul 21;66(4):549–63. doi: 10.1007/s00267-020-01328-y (PMC7522067; doi:10.1007/s00267-020-01328-y)
Supplement: Supplementary file 1 — Appendix [file 267_2020_1328_MOESM1_ESM.docx]

**Appendix**

Table A1. Means, standard deviations, and alpha for the psychological predictors.

|  | Means (Standard deviation) | Alpha, α |
| --- | --- | --- |
| Actual knowledge |  |  |
| Declarative objective knowledge scale (DEC-OBJ)^a^ | 3.74 (1.37) | na |
| Procedural objective knowledge Production  (PROC-OBJ PROD)^b^ | 2.35 (0.78) | na |
| Procedural objective knowledge Biodiversity  (PROC-OBJ BIO)^b^ | 2.27 (0.90) | na |
| Procedural objective knowledge Recreation  (PROC-OBJ REC)^b^ | 2.35 (0.78) | na |
| Procedural objective knowledge Adaptation  (PROC-OBJ ADAPT)^b^ | 2.78 (0.87) | na |
| Procedural objective knowledge Mitigation  (PROC-OBJ MIT)^b^ | 2.34 (0.77) | na |
| Confidence^c^ |  |  |
| Declarative subjective knowledge (DEC-SUBJ) | 2.71 (0.71) | na |
| Procedural subjective knowledge: Production  (PROC-SUBJ PROD) | 2.80 (0.76) | na |
| Procedural subjective knowledge: Biodiversity  (PROC-SUBJ BIO) | 2.55 (0.74) | na |
| Procedural subjective knowledge: Recreation  (PROC-SUBJ REC) | 2.48 (0.76) | na |
| Procedural subjective knowledge: Adaptation  (PROC-SUBJ ADAPT) | 2.06 (0.73) | na |
| Procedural subjective knowledge: Mitigation  (PROC-SUBJ MIT) | 2.32 (0.74 | na |
| Basic values^d^ |  |  |
| Openness to change (OC) | 5.01 (1.04) | .74 |
| Conservation (C) | 5.49 (0.91) | .70 |
| Self-enhancement (SE) | 3.44 (1.21) | .77 |
| Altruistic (A) | 5.33 (1.11) | .77 |
| Biospheric (B) | 5.32 (1.20) | .83 |
| Forest values^e^ |  |  |
| Production forest values (PROD VALUE) | 5.41 (1.35) | .77 |
| Biodiversity forest values (BIO VALUE) | 5.27 (1.33) | .88 |
| Recreation forest values (REC VALUE) | 4.93 (1.48) | .89 |
| Forest owner identity^f^ |  |  |
| Production/private forest owner identity  (PROD PRIVATE FOI) | 2.77 (0.84) | .69 |
| Consumption/public forest owner identity  (CON PUBLIC FOI) | 3.56 (0.75) | .62 |
| Social forest owner identity (SOCIAL FOI) | 2.51 (1.08) | .69 |
| Distant forest owner identity (DISTANT FOI) | 1.82 (0.70) | .65 |
| Central forest owner identity (CENTRAL FOI) | 3.90 (0.80) | .77 |

^a^ Scale 0-6, where a higher value represents a higher level of declarative objective knowledge.

^b^ Scale 0-4, where a higher value represents a higher level of procedural objective knowledge.

^c^ Scale 1-4 (1 = No knowledge at all, 2 = A little knowledge, 3 = Certain knowledge, 4 = Extensive knowledge).

^d^ Scale -1-7 (-1 = Opposed to my values, 0 = Not important, 3 = Important, 6 = Very important, 7 = Extremely important).

^e^ Scale 1-7 (1 = Not at all important, 7 = Very important).

^f^ Scale 1-5 (1 = Totally disagree, 5 = Totally agree).

Na = not applicable.

Table A2. Statements measuring forest owner identity (FOI) (including self-identity, social identity, and centrality).

| Concept | Measures (questions and statements) | |
| --- | --- | --- |
|  | Self-identity: | Social identity: |
|  | “To what extent do you agree with the following statements by you as a forest owner?”  Five-point scale (1 = Totally disagree, 5 = Totally agree). | “To what extent do you identify with other forest owners who…:”  Five-point scale (1 = Totally disagree, 5 = Totally agree). |
| Production/private FOI (n = 5) | I perceive my forest to be an economic asset to be used whenever needed.  I buy and sell forest.  I use economic calculations when I make decisions about my forest.  I manage my forest in terms of a family heritage.* | manage their forest for future needs?  use their forest to earn money? |
| Consumption/public FOI (n = 6) | I use my forest for hunting.  I manage my forest so that others would like to visit it.  I would also like future generations in Sweden to benefit from my forest.  I place great emphasis on promoting nature values in my forest.  I use resources from my forest for personal use (e.g., firewood, berries). | use their forest for personal needs or interests (e.g., hunting, recreation)?  put the public’s interests concerning the forest above their own?* |
| Social FOI (n = 3) | I discuss forest management with other forest owners.  I take part in a network of forest owners. | collaborate with forest owners? |
| Distant FOI (n = 6) | I don’t pursue any active management in my forest.  I allow others (e.g., relatives, a manager) to make decisions about how my forest should be managed.  I do not take part in decisions concerning my forest.  I let my forest manage itself. | leave decisions about forest management to others?  are passive? |
|  |  |  |
| Centrality (n = 6) | “To what extent do you agree with the following statements about you and your forest?”  Five-point scale (1 = Totally disagree, 5 = Totally agree).  I love my forest.  My forest makes me unhappy. (Reversed)  My forest is part of who I am  I feel no personal attachment to my forest (Reversed)  My forest is one of my most important possessions  Forest ownership is an important part of my life | |

n = number of items. *Item excluded.

Table A3. Management strategies.

| Production | Cleared and thinned at the optimal time to increase the forest’s economic value.  Conducted final felling when the forest reached optimal felling age.  Conducted soil scarification to improve the preconditions for rejuvenation.  Fertilized to improve growth. |
| --- | --- |
| Biodiversity | Left dead wood and debris in the forest.  Used continuous cover forestry.  Avoided forest management measures to increase environmental values in the forest.  Left tall stumps during final felling and thinning. |
| Recreation | Cleared or thinned to open up paths.  Cleared or thinned to create clearings.  Taken special consideration when using forestry machines to make sure paths are not damaged.  Cleared or thinned to accentuate deciduous trees, thereby making the forest more beautiful where people move about. |
| Climate adaptation | Increased the share of mixed stand with both coniferous and deciduous trees during clearing, thinning or rejuvenation.  Increased the share of deciduous stand during clearing, thinning or rejuvenation.  Implemented site-adapted forestry (i.e., more carefully selected the right tree species on the right land).  Increased the variation in felling age and thinning program. |
| Climate mitigation (substitution) | Employed advance felling (shorter rotation) to allow forest raw material to replace products causing more emission of carbon dioxide.  Harvested branches and crowns for bio-fuel production.  Broken stumps for bio-fuel production.  Postponed felling (longer rotation) to allow forests to grow for a longer period of time and thereby store more carbon.* |

Five-point scale (1 = never, 2 = seldom, 3 = sometimes, 4 = often, 5 = always).

*Item excluded.
